# Oral Health and Older Adults: A Narrative Review

**DOI:** 10.3390/dj12020030

**Published:** 2024-02-01

**Authors:** Martin S. Lipsky, Tejasvi Singh, Golnoush Zakeri, Man Hung

**Affiliations:** 1 College of Dental Medicine, Roseman University of Health Sciences, South Jordan, UT 84095, USA; 2 College of Urban and Public Affairs, Portland State University, Portland, OR 97201, USA; 3 Division of Public Health, University of Utah, Salt Lake City, UT 84108, USA

**Keywords:** oral health, older adults, elderly, general health

## Abstract

Oral health’s association with general health, morbidity, and mortality in older adults highlights its importance for healthy aging. Poor oral health is not an inevitable consequence of aging, and a proactive, multidisciplinary approach to early recognition and treatment of common pathologies increases the likelihood of maintaining good oral health. Some individuals may not have regular access to a dentist, and opportunities to improve oral health may be lost if health professionals fail to appreciate the importance of oral health on overall well-being and quality of life. The authors of this narrative review examined government websites, the American Dental Association Aging and Dental Health website, and the Healthy People 2030 oral objectives and identified xerostomia, edentulism, caries, periodontitis, and oral cancer as five key topics for the non-dental provider. These conditions are associated with nutritional deficiencies, poorer quality of life, increased risk of disease development and poorer outcomes for cardiovascular disease, diabetes, and other systemic conditions prevalent among older adults. It is important to note that there is a bi-directional dimension to oral health and chronic diseases, underscoring the value of a multidisciplinary approach to maintaining oral health in older adults.

## 1. Introduction

By 2030, the elderly population will increase so that one in every five residents, or more than 70 million Americans, will be 65 years or older [1]. As the population ages, so will the burden of chronic disease. Among the most common diseases affecting older adults are chronic diseases of the oral cavity, including dental infections (e.g., caries, periodontitis), tooth loss, mucosal lesions, and oral cancer. These conditions can adversely affect nutrition, self-esteem, quality of life, and general health [2]. Oral health’s association with general health [3], morbidity, and mortality in older adults highlights its importance for healthy aging.

Despite its importance, poor oral health remains a common morbidity for older individuals. While improvements in dental treatments, community water fluoridation, and better oral hygiene have improved dentition, age-related changes in physiology, comorbidities, and polypharmacy make older adults more vulnerable to oral diseases [4]. Issues such as accessibility, cognitive impairment, and disability add to the vulnerability of this demographic. More than 60% of seniors lack dental insurance [5]. Not surprisingly, a high percentage of older adults have unmet dental needs and do not see a dentist annually [6,7].

Poor oral health is not an inevitable consequence of aging, and early recognition of common pathologies increases the likelihood of maintaining good oral health throughout a lifetime. Some patients who do not visit a dentist will see a non-dental provider, presenting opportunities to improve oral health. Oral diseases and many chronic systemic diseases share common risk factors such as unhealthy diet, tobacco use, and alcohol consumption that can benefit from education outside a dental office. The increasing body of evidence documenting a bi-directional association between oral health and chronic systemic disease indicates the importance of incorporating oral health into chronic disease management strategies. Given the inseparable linkages between oral and systemic health, it is not surprising the American Dental Association recommends a multi-disciplinary approach to oral health. However, gaps in knowledge and awareness of the importance of oral health among health professionals may present barriers to optimizing oral health [8,9,10,11].

This narrative review sought to review and synthesize the literature into a cohesive summary of the current knowledge related to oral health and older adults to increase awareness of its importance and address gaps in non-dental providers’ knowledge. These aims should help busy non-dental providers seeking up-to-date information about oral health to develop strategies that improve the oral health of their patients.

## 2. Methodology

To organize and synthesize articles related to the narrative review topic of oral health and the elderly, two authors (MSL, MH) independently reviewed government websites, the American Dental Association Aging and Dental Health website, and the Healthy People 2030 oral objectives. To determine what topics to include in this narrative review, each author (MSL, MH) developed a list of key topics about oral health and older adults. The group then discussed these to achieve consensus agreement about the five most important topics for the non-dental healthcare provider. The five identified topics included xerostomia, edentulism, caries, periodontitis, and oral cancer.

A narrative review search strategy was used to locate relevant literature on current knowledge related to oral health and older adults. For this review, the authors (MSL, MH, GZ, TS) searched PubMed from 2003 to 2023 using the MeSH terms “xerostomia,” “tooth loss,” “caries, root,” “caries, dental,” “periodontal disease,” “dental disease,” “oral cancer” and “frail older adults.” Google Scholar was also searched from 2003 to 2023 using these terms: oral health, periodontal disease, caries, dental disease, edentulism, tooth loss, xerostomia, and older adults, where the first 10 pages of results returned by the search engine were examined. Retrieved articles were reviewed by the study team for topic relevance. The bibliographies of identified manuscripts were also reviewed for additional articles of relevance and other data sources potentially relevant to this review. When appropriate, governmental websites such as the Centers for Disease Control and Prevention and the National Institutes of Health were used as statistical and epidemiologic data sources. The authors (MSL, MH, GZ, TS) considered all types of peer-reviewed and full-length studies in English, which included randomized controlled trials (n = 5), clinical trials (n = 5), observational studies (n = 56), retrospective studies (n = 15), meta-analyses (n = 1), systematic review articles (n = 4), and other types of reviews (n = 41). For the guiding principles of this review, we used a critical appraisal tool called SANRA (Scale for the Assessment of Narrative Review Articles) to control for the quality of the review process regarding findings in this manuscript [12]. SANRA covers the following topics: (1) explanation of the review’s importance/relevance, (2) statement of the aims of the review, (3) description of the literature search, (4) targeted referencing, (5) solid logic or scientific reasoning, and (6) adequate presentation of relevant and appropriate endpoint data, also with conclusions.

The value of a narrative literature review is that it can provide an overview of a content area or subject. While this type of review typically employs a non-systematic review strategy, it still plays a vital role by providing readers with up-to-date knowledge about a specific topic [13]. For healthcare professionals, a narrative review can efficiently summarize large amounts of information about a patient care topic issue into just a few pages [14].

## 3. Xerostomia

Xerostomia is the sensation of oral dryness characterized by a reduction in salivary flow and alterations in saliva composition [15]. While the subjective complaint of dry mouth and xerostomia are sometimes used interchangeably, true xerostomia is the consequence of acute or chronic salivary gland hypofunction with inadequate salivary secretion. Typically, patients complain of oral dryness when the salivary secretion is reduced by >50%. However, the sensation of dry mouth can also occur despite the normal secretory function of the salivary glands [16], a condition known as pseudo-xerostomia or false xerostomia [17]. Causes of this subjective symptom include changes in the composition of saliva [16], mouth breathing, atypical oral and facial symptoms, burning mouth syndrome, oral dysesthesia, and mental, psychological, and psychiatric disorders. In over half of false xerostomia cases, a 50% decrease in the amount of oral fluids was observed [18].

Various age-related factors, including changes in salivary gland structure and function, medication use, systemic diseases, and psychosocial aspects, contribute to xerostomia in older individuals [19]. As many as one in two older adults in a primary care setting experience some degree of dry mouth, with a higher prevalence among females [19,20]. Medication plays a pivotal role in the increased prevalence as older adults are more likely to be prescribed multiple medications, many of which can induce xerostomia as a side effect [21].

The diagnosis of xerostomia is primarily based on history and physical examination. Findings may include a lack of pooled saliva, sticky mucous membranes, reddened mucosa, and a loss of tongue fissuring and papilla. If necessary, sialometry is a diagnostic tool that can objectively measure salivary flow rates and assess salivary gland function [19].

The most frequently reported cause of xerostomia is xerostomic drugs [22]. These medications induce xerostomia by directly suppressing the production of acetylcholine or blocking either muscarinic or adrenergic receptors [23]. Other causes of xerostomia include head and neck radiation, which can damage salivary glands, Sjogren’s syndrome, and autoimmune diseases, such as SLE, which affect salivary gland function [24,25].

While often thought of as merely a nuisance, xerostomia exerts a substantial impact on affected individuals, influencing not only their oral health but also their social and emotional life. A dry mouth can lead to difficulties speaking, chewing, and swallowing, diminishing a person’s overall quality of life [19]. It also increases the susceptibility to dental caries, periodontal disease, halitosis, and candidiasis. Psychosocial factors, including emotional stress and anxiety, can exacerbate the sensation of dry mouth in elderly individuals and compound the impact of xerostomia [26]. Xerostomia also contributes to altered oral sensation among cancer patients, which can adversely affect their relationship with food, quality of life, and overall health outcome [27].

Treatment objectives for xerostomia can generally be categorized into three primary areas: increasing oral moisture and salivary flow, addressing the underlying systemic condition, and preventing tooth decay [28]. Xerostomia associated with systemic diseases requires optimizing care for the underlying cause, educating patients about hydration, and avoiding triggers like tobacco, coffee, alcohol, and hard-to-chew foods [29]. Promoting optimal oral hygiene and dental care minimizes dental complications associated with reduced salivary flow.

Strategies to improve oral moisture include increasing fluid intake, saliva substitutes, xylitol gum, sucking on mints or candy, and adjusting or discontinuing xerogenic medications [21]. Saliva substitutes come in several forms, including sprays, lozenges, and gels, that may be used before meals and as needed. Unfortunately, they can be expensive and are variably effective with a limited duration of action. Since medications often affect salivary gland function, reviewing a patient’s medication list and discontinuing or switching to a drug with less effect on saliva flow should be considered. Anticholinergic and antidepressant drugs are among the most common offending agents, but more than 500 drugs can affect salivary gland function [30]. Table 1 outlines drug classes associated with dry mouth and examples of offending agents from each class.

Two FDA-approved drugs that stimulate salivary flow are cevimeline and pilocarpine [31,32]. Both work on muscarinic receptors to stimulate salivation but require some residual salivary gland function to work and need up to a three-month trial to determine their efficacy [33]. Side effects include sweating, nausea, and rhinitis. Topical physostigmine is an alternative option that may have fewer side effects [34]. While pharmaceutical interventions can provide relief, it is essential to include their use as part of a comprehensive approach.

Adjunct aids also play a pivotal role in managing the adverse consequences of xerostomia. These may include implementing a low-sugar diet to reduce the risk of dental caries [32], appropriate fluoride supplementation and fluoride mouthwash to strengthen enamel. Antimicrobial mouthwashes can aid in maintaining oral hygiene and preventing infections in individuals with compromised salivary function [35]. Table 2 summarizes the management of xerostomia.

## 4. Edentulism

Edentulism, or the complete loss of natural teeth, is a significant concern for overall well-being. Edentulism can be the result of congenital, iatrogenic, or traumatic causes, but dental caries and periodontal diseases are the main causative factors of teeth loss in the elderly [36]. Edentulism often represents the final stage of untreated caries or periodontal disease [37]. Although the prevalence of complete tooth loss has declined due to improved oral health care and access to dental services, edentulism remains a major problem for older adults. About one in six older adults (17%) in the US have lost all their teeth. Among older adults who are current smokers, 43% had lost all of their teeth, which is more than three times the prevalence among those who never smoked (12%) [38]. Women are also more susceptible to edentulism [39]. In contrast to the declining rates of tooth loss seen in developed countries, some note an opposite trend in developing nations [40,41].

Studies link several behavioral risk factors, such as tobacco use, consumption of alcoholic beverages, and a poor diet, to edentulism [42]. Additionally, systemic conditions such as diabetes, cardiovascular disease, hypertension, and endocrine disorders can contribute to tooth loss in the elderly [43]. Socioeconomic factors, including low income, no dental insurance, minimal education, and limited access to dental care, are also associated with an increased prevalence of edentulism [44,45].

Addressing edentulism is important because of its harmful effects on oral and general health. Oral consequences include mandibular bone loss, impaired masticatory function, an unhealthy diet, social disability, and poor quality of life. Denture use is associated with denture stomatitis, an inflammatory condition of the palatal mucosa resulting in redness, swelling, and tenderness [46]. It occurs more commonly when dentures are not properly fitted, are not removed nightly, or when dentures are not cleaned properly. Wearing dentures alters the oral microbiome, and about 90% of dental stomatitis is due to an overgrowth of candida [47]. Good denture hygiene is the most important aspect of treatment and includes removing dentures at night, properly cleaning and disinfecting dentures, and storing them overnight in an antiseptic solution. Often, a topical antifungal agent and antimicrobial mouthwash such as chlorhexidine are prescribed [46]. Cases that fail to respond to the usual treatments suggest the possibility of systemic diseases, such as type 2 diabetes mellitus, that compromise the ability to fight infection [48].

Edentulous individuals are also at risk for nutritional compromise. For every five teeth lost, a person is 1.42 times more likely to have a decreased intake of vital nutrients and is also at greater risk for obesity [49]. Other comorbid conditions associated with edentulism include cardiovascular disease, rheumatoid arthritis, pulmonary diseases (including chronic obstructive pulmonary disease), and cancer. Not surprisingly, a reduced number of natural teeth that are not restored increases the risk of mortality [49].

Managing edentulism requires a comprehensive approach. Removable dentures, implant-supported fixed prostheses, and implant-supported dentures are among the available treatment options [50]. Individualized treatment plans tailored to each patient’s unique needs and preferences are crucial in achieving successful outcomes and improving their quality of life [51]. Regular tissue check-ups are essential to ensure the health and stability of oral prostheses and prevent complications [52]. Moreover, ongoing patient education is paramount since it empowers elderly individuals to maintain good oral hygiene practices [53].

## 5. Caries

Caries is a very prevalent oral health problem [54]. Commonly known as tooth decay or cavities, caries is a dental condition characterized by the demineralization of the tooth structure due to the action of cariogenic bacteria metabolizing sugars to produce acid [55]. This acid attacks and damages the tooth surface, leading to caries formation [55]. Key factors contributing to caries development include the presence of cariogenic bacteria, consumption of sugary drinks, high-carbohydrate diets, and inadequate oral hygiene practices [56]. Due to age-related salivary and immune function changes, multiple co-morbidities, and medication-induced xerostomia, caries are the most frequent dental pathology encountered among the elderly [57]. Gingival recession, which is common as individuals age, makes root caries more common among older adults.

The relationship between systemic diseases and caries is significant, with several medical conditions, such as diabetes and polypharmacy, associated with dental caries. Xerostomia greatly increases the risk of caries [58]. Maintaining oral health is particularly crucial for geriatric populations, as more adults are retaining their natural teeth, emphasizing the importance of preventive measures [59].

Management of dental caries includes both preventive and treatment strategies. Preventive measures are essential for mitigating caries risk and include regular semi-annual dental visits, professional cleanings, and fluoride use [60]. Proper at-home oral hygiene practices play a pivotal role in reducing the prevalence of caries, particularly among the elderly population. Educating individuals about dietary choices, the importance of regular dental visits, and the correct use of fluoride products can empower them to take proactive steps to preserve their oral health [61].

Fluoride remains a cornerstone in caries prevention, with daily fluoride concentration recommendations proving highly effective in remineralizing teeth. For older populations, especially those in institutionalized settings, nursing homes, or home-bound adults, the use of higher fluoride toothpaste and fluoride varnish concentrations is recommended over conventional toothpaste [61]. Daily use of a 0.2% NaF mouthwash also reduces caries risk [62].

Research supports the use of fluoride toothpaste (5000 ppm) twice daily and monthly fluoride varnish application as effective strategies for reducing caries risk [63]. Additionally, for individuals with root caries, the application of 5% NaF four times a year and yearly application of silver diamine fluoride have been proposed as beneficial interventions [64]. While fluoride is well-accepted as a tool for reducing caries, a systematic review highlights the need for further research on the best strategies for fluoride use in caries prevention among older adults [60]. Some additional products, such as chlorhexidine, xylitol, and casein phosphopeptide-amorphous calcium phosphate, also reduce cariogenic bacteria, but to date, studies do not provide conclusive evidence of their benefit in arresting caries development or tooth remineralization.

Dental caries merit treatment to control pain and to prevent tooth loss, which adversely affects aesthetics, chewing efficiency, speech, and social interaction. Untreated caries may also progress to irreversible pulpitis, tooth loss, and abscess formation, which can spread to surrounding tissues. Symptoms include thermal sensitivity and mild-to-severe pain. Overall, about one in five individuals over age 65 have dental decay that needs treatment [65]. As people live and retain their teeth longer, it is likely there will be an increase in untreated caries in this growing demographic [66].

Traditional treatment involves surgical intervention and restoration [67]. Advanced dental caries may necessitate root canal treatment or extraction. Optimizing treatment should be individualized and incorporate factors such as overall health, decisional capacity, cognition, physical disability, and personal preference. Silver diamine fluoride (SDF) application can arrest cavities and is emerging as a cost-effective nonsurgical option for older individuals with medical, physical, or mental issues that make restoration challenging or for those who have difficulties affording dental care [68,69,70]. While safe and effective, a disadvantage is that SDF turns the decayed area permanently black.

## 6. Periodontitis

Periodontal disease encompasses gingivitis and periodontitis and is characterized by bacterial infection leading to gingival inflammation, tooth loss, bone resorption, and gingival recession [66]. Gingivitis is the earliest stage of periodontal disease and is used to describe inflammation between the gingival line and tooth. Gingivitis can often be reversed with improved oral hygiene [71,72,73,74]. Periodontitis occurs when the microbially induced, host-mediated inflammation progresses into a chronic, destructive, irreversible disease state that damages the tooth attachment and the supporting bone [75]. Tooth loss and edentulism represent the final stages of untreated periodontitis [75].

Epidemiological studies note the greater loss of attachment with increasing age, and by age 65, about 70% of individuals have periodontitis [76,77]. Some evidence suggests that age-related changes in the immune system, cellular senescence and “inflammaging”, and impaired wound healing play key roles in the pathogenesis of periodontal disease [76]. The risk factors for periodontitis in older adults are like those in younger age groups and include inadequate brushing and flossing, being poorer, less educated, non-insured and cigarette smoking [75].

Among the aging population, systemic diseases have been identified as accelerators of periodontal disease progression. Diabetes mellitus, respiratory diseases, cardiovascular disease, stroke, osteoporosis, arthritis, and Alzheimer’s disease have all been linked to an increased risk of periodontal disease [66,78]. Evidence also suggests there is a bi-directional link between periodontitis and systemic disease, with associations established for periodontitis and an increased risk for several chronic diseases, including cardiovascular diseases [79], diabetes, rheumatoid arthritis, cancer, and chronic obstructive pulmonary disease [80,81].

Tobacco use is the most important modifiable risk factor, and smoking cessation reduces the risk of periodontitis and tooth loss [82]. Moreover, over half of adults 65 years and older report taking four or more prescription drugs to manage chronic conditions, and some drugs, including those for cardiovascular diseases and diabetes, cause xerostomia, which can exacerbate periodontal issues [83,84].

Gingival swelling, redness, and bleeding when brushing or flossing suggest periodontal disease [76]. A dentist establishes the diagnosis of periodontal disease by using a probe to assess bleeding and by measuring the depth of the periodontal pocket or gap between the gingival and the tooth and how far down it is until the gingival attaches to the tooth [85]. Probing depths greater than 3 mm indicate periodontal disease, and these pockets serve as a breeding ground for bacterial pathogens. Studies demonstrate that high levels of specific bacteria, including Porphyromonas gingivalis, Tannerella forsythia, Treponema denticola, and Fusobacterium nucleatum, are detected when periodontal disease is present [86]. In addition to the clinical exam, radiographs are indicated for individuals with clinical evidence of periodontal destruction to assess alveolar bone loss.

Restorative considerations, such as indirect restorations, crowns, bridges, partial dentures, and implants, potentially increase plaque accumulation and caries risk, necessitating extra measures in these patients to mitigate bacterial growth in the periodontium [87]. This is particularly relevant to older adults who require frequent dental restorations to maintain oral function and aesthetics.

Prevention strategies for periodontal disease involve controlling co-morbidities and, when necessary, restorative treatments to maintain oral health [76]. Dentists should assess and stage the degree of periodontitis and incorporate the medical history when formulating treatment plans. Chlorhexidine mouthwash, a broad-spectrum oral antimicrobial rinse, helps limit plaque formation, although its long-term use may lead to tooth staining [88]. Chlorhexidine has both a bactericidal action and a prolonged bacteriostatic action due to absorption into the enamel. Chlorhexidine also comes in a wafer form that can be directly inserted into the periodontal pocket. Electric toothbrushes, interdental brushes, water flossers, and oral rinses are also recommended to reduce the microbial burden in the oral cavity [89].

Regular dental check-ups every six months to assess hygiene and home care techniques, monitor probing depths, and detect bone loss are essential in the management of periodontal disease [84]. Additionally, comprehensive health check-ups can help identify and address underlying systemic conditions that may contribute to gingival disease. It is crucial to recognize that periodontal disease can have a significant impact on both life expectancy and quality of life if not promptly assessed and managed in older individuals.

## 7. Oral Cancer

Oral cancer (OC) is the most common form of head and neck cancer and includes cancers of the lips, tongue, palate, oropharynx, tonsils, and other oral structures. The most common type is squamous cell carcinoma. OC is a disease of older adults with an average age of 64 years at the time of diagnosis. Each year, there are over 54,000 new cases and 11,000 deaths from oral cancer [90].

The non-dental provider’s role consists primarily of prevention and early diagnosis. About three out of four cases occur in individuals with one or more risk factors, and counseling patients about these risks offers an opportunity for primary prevention [91]. Tobacco use is the strongest risk factor, and smoking tobacco and smokeless tobacco products both increase the risk of OC [92]. Alcohol, particularly heavy drinking, also increases the risk of oral cancer. Smoking and alcohol act synergistically, and individuals who both smoke and drink increase their OC risk by as much as 30 times more than nonsmokers and drinkers [93].

OC is also associated with the human papillomavirus (HPV) [94]. While the HPV vaccine protects against several types of HPV linked to oral cancer, the vaccine first became available in 2006 [94]. Since cancer takes years to develop, a significant decline in OC from HPV vaccination may not become evident until the 2040s [95]. While not a common practice in the US, chewing betel nut is endemic in large parts of Asia and increases the risk of OC.

Early diagnosis improves outcomes. The principal means for an early diagnosis is a physical examination that consists of systematic inspection and palpation. The 5-year survival rate for an early-stage OC is over 80%, but only about a quarter of cases are diagnosed at an early stage [96]. Once the cancer spreads to the surrounding tissues or the regional lymph nodes, the 5-year survival rate declines, and for individuals presenting with distant metastases, the 5-year survival falls to 40% [97]. An awareness of the common presenting symptoms, such as a non-healing lesion, red or white patches, and hoarseness, is key to making an early diagnosis. A thorough visual inspection of the entire oral cavity, digital palpation, and lymph node assessment detects most OCs [98]. Removing precancerous lesions, such as leukoplakia or erythroplakia, reduces the incidence and mortality of OCs. Leukoplakia are white patches that do not wipe off and cannot be characterized clinically or pathologically. Erythroplakia is the red counterpart to leukoplakia and refers to a red patch of tissue without an obvious cause. When scraped, erythroplakia tends to bleed easily. Squamous cell carcinomas are usually present as a non-healing ulcer or mass. Consequently, suspicious lesions, growths and non-healing ulcerations merit referral for evaluation and biopsy. Lesions that appear benign and relate to a reversible condition, such as local irritation or infection, can be treated and reassessed in 10 to 14 days [99]. Failure to improve after 2 to 3 weeks indicates the need for additional testing [99].

## 8. Implications

The American Dental Association advocates a multidisciplinary approach to maintaining oral health in older adults. Individuals who visit a primary care provider may not have a dentist, creating opportunities for other health professionals to improve oral health through education and appropriate referrals. Early recognition of dental disease and referral increases the likelihood of individuals retaining their natural teeth, which can impact nutrition, self-esteem, and overall health. A multidisciplinary approach recognizes a growing body of evidence linking oral health with several chronic diseases that are prevalent among older adults, including diabetes, cardiovascular diseases, rheumatoid arthritis, Alzheimer’s disease, and Parkinson’s disease [3,100]. These systemic diseases and their related medications place older adults at greater risk for oral health conditions, such as periodontal disease, dental caries, and even oral precancerous and cancerous lesions. Often overlooked is that there is a bi-directional dimension to oral health and chronic disease. Studies demonstrate that addressing oral pathology can improve blood pressure and blood sugar [101,102]. Medications can adversely affect oral health, and involving pharmacists along with dentists and primary care providers can help develop comprehensive care plans tailored to the unique needs of each patient [103].

## 9. Conclusions

Xerostomia, edentulism, caries, periodontal disease, and oral cancer represent five common oral pathologies among elderly patients. These conditions are associated with nutritional deficiencies, poorer quality of life, increased risk of disease development and poorer outcomes for cardiovascular disease, diabetes, and other systemic conditions prevalent among older adults. Opportunities to improve oral health may be missed by health professionals who may fail to appreciate the importance of oral health on overall well-being and quality of life.

## Figures and Tables

**Table 1:** Classes and characteristics of drugs.

Class	Examples	Comment
Anticholinergic Agents	oxybutynin benztropine mesylate atropine bronchodilators, e.g., albuterol formoterol anti-migraine medications, e.g., zolmitriptan rizatriptan donepezil	Blocks ACH, a neurotransmitter that stimulates saliva flow
Antidepressants, antipsychotics	SSRIs e.g., citalopram haloperidol phenelzine amitriptyline imipramine bupropion SNRIs, e.g., venlafaxine	Effect on neurotransmitters can interfere with salivary gland function
Diuretics	furosemide chlorothiazide hydrochlorothiazide	Disrupts fluid balance
Antihypertensive Agents	captopril lisinopril enalapril beta-blockers, e.g., metoprolol timolol clonidine alpha- blockers, e.g., prazosin terazosin calcium channel blockers	May affect salivary glands indirectly through their impact on blood pressure regulation and anti-cholinergic effects
Sedative and Anxiolytic Agents	alprazolam diazepam triazolam lorazepam antipsychotics, e.g., phenothiazines risperidone trifluoperazine quetiapine non-benzodiazepene hypnotics, e.g., zolopdem eszopiclone zolpiclone	Depressant effect on the central nervous system, potentially leading to reduced salivary flow rates
Muscle Relaxants	tizanidine cyclobenzaprine orphenadrine	May effect nerve signaling
Analgesic Agents	opioid medications, e.g., morphine codeine NSAIDs	Sympathomimetic action, which impairs salivary gland function
Antihistamines	astemizole clemestine loratadine fexofenadine brompheniramine cetirizine diphenhydramine	Has anti-cholinergic activity
Stimulants	dextroamphetamine dextroamphetamine/amphetamine methylphenidate	Sympathomimetic action, which impairs salivary gland function
Bronchodilators	albuterol fomoterol fluticasone	Impair salivary gland function

ACH = acetylcholine; NSAIDs = nonsteroidal anti-inflammatory drugs.

**Table 2:** Management of xerostomia.

Treatment Strategies	Comment
Non-pharmacologic	Includes hydration, lozenges, chewing xylitol gum
Medication Review	Identify possible offending agent and, if possible, switch to a drug with less impact on salivation
Health Habits	Limit coffee, no tobacco use
Oral Hygiene	Regular dental checkups and cleaning, good home oral hygiene practices
Saliva substitute	Comes in sprays, lozenges, and gels that may be used before meals and as needed. Effect limited in duration, may alter taste, and can be expensive
Cevimeline	Stimulates muscarinic receptors. Dose 30 mg tid. May have fewer cardiac and/or pulmonary side effects than pilocarpine. Common side effects: nausea, headache, sweating
Pilocarpine	5 mg three to four times a day. Common side effects: nausea, headache, sweating
Physostigmine Gel	Physostigmine in gel applied to the inside of the lips and distributed with the tongue was superior to placebo

## Data Availability

Data are contained within the article.
